# Tolerability of duloxetine in elderly and in non-elderly adults: a protocol of a systematic review and individual participant data meta-analysis of randomized placebo-controlled trials

**DOI:** 10.1186/s13643-022-01945-0

**Published:** 2022-04-15

**Authors:** Jean-Charles Roy, Chloé Rousseau, Alexis Jutel, Florian Naudet, Gabriel Robert

**Affiliations:** 1 Adult Psychiatry Department, Rennes University Hospital, 2 avenue du Professeur Léon Bernard, 35042 Rennes, France; 2 Clinical Investigation Center INSERM 1414, Department of Clinical Pharmacology, Rennes University Hospital, Rennes, France

**Keywords:** Psychiatry, Geriatric, Meta-analysis, Drug safety, Duloxetine hydrochloride

## Abstract

**Background:**

Duloxetine is an antidepressant that benefits from a wide range of approval in the elderly population, while its safety for use compared to non-elderly is not clearly assessed. This protocol outlines a systematic review and individual participant data meta-analysis comparing the tolerability of duloxetine between elderly and non-elderly.

**Methods:**

Searches will be conducted in PubMed, ClinicalTrials.gov, Clinicaltrialsregister.eu, data sharing platforms, FDA drug approval packages, European public assessment reports and withdrawn applications from the EMA website. The review will be performed on studies available in electronic databases from their date of inception to the 31 March 2022. Only randomized controlled clinical trials, comparing duloxetine to placebo, will be included in this meta-analysis. The studies will be selected if they comprise both elderly and non-elderly adults, in conditions of use of duloxetine approved by the European Medical Agency (EMA) and the Food and Drug Administration (FDA). The primary outcome will be the rate ratio of serious adverse events under duloxetine compared to placebo, between participants at least 65 years old and non-elderly. Second, the number of any adverse events, clinical efficacy and quality of life will be compared between elderly and non-elderly under both interventions. The quality of evidence in the tolerability of duloxetine will be assessed using the GRADE system. A one or two-stage individual participant data random effect meta-analysis will be conducted depending on the availability of the data.

**Discussion:**

This meta-analysis will investigate the tolerability safety of duloxetine in the elderly population across all conditions approved by European and American regulatory authorities. The results from this meta-analysis are intended to help prescribers to provide better care for the elderly population.

**Systematic review registration:**

The protocol has been registered at the International Prospective Register of Systematic Reviews (PROSPERO; registration number: CRD42019130488).

**Supplementary Information:**

The online version contains supplementary material available at 10.1186/s13643-022-01945-0.

## Background

### Rationale

Duloxetine is a medication that has been approved for a wide range of pathologies by the Food and Drug Administration (FDA) and the European Medicines Agency (EMA), namely, major depressive disorder, generalized anxiety disorder, diabetic peripheral neuropathic pain, fibromyalgia, chronic musculoskeletal pain and stress urinary incontinence. These conditions represent a major public health burden for the elderly population [1]. While several guidelines recommend the use of duloxetine as a first-line medication for these conditions in the elderly [2–6], others formally proscribe it [7], making the prescription of duloxetine in the elderly difficult to assess for physicians.

This heterogeneity in good practice guidelines is in part due to the mixed evidence for a favourable risk-benefit balance in older populations [5, 8]. For instance, a mixed treatment meta-analysis, mostly based on indirect evidence, suggests that duloxetine had higher response rates than other antidepressants for major depressive disorder in the old-age population (≥ 65 years old), in the context of scarce data [9]. In another meta-analysis from three trials in elderly population, duloxetine was found to be associated with high rates of response in depression but with increased risk of adverse events [10]. In non-elderly, several meta-analyses concluded that duloxetine was associated with a disfavouring risk-benefit balance compared to placebo in osteoarthritis and stress urinary incontinence [11, 12]. Meta-regression analyses from the systematic evaluation of safety and efficacy of 21 antidepressants by Cipriani et al. (2018) found similar results, observing a significant association between the mean age of participants and response to duloxetine (odds ratio (OR) = 1.86, confidence interval (CI) 95% = 1.66–2.08) with an association between age and number of drop-outs under duloxetine (OR = 1.10, CI 95% = 0.97–1.24) [13], although the latter result did not reach statistical significance.

Early premarketing pharmacokinetic studies provide further concerns on the safety of use of duloxetine in the elderly. Lilly’s study SAAY concluded a reduced oral clearance of duloxetine of 25% in an elderly class (mean age of 69 years old) compared to a middle-aged class (mean age of 42 years old) [14] of women under 40 mg of duloxetine. Furthermore, the area under the curve measure of exposure was found to be 24% higher in elderly individuals, although it was not statistically significant in the cohort of 24 participants, but with half-life elimination reduced by one-third in elderly individuals compared to that in middle-aged individuals. A statistically significant correlation of diminished oral clearance of duloxetine with age was demonstrated, which was estimated to be a reduction in clearance of almost 60% in individuals between 23 and 76.8 years old (Study SAAB) [14]. Skinner et al. (2004) estimated that a decrease in oral clearance accelerates at approximately 52 years of age [15]. Similar results have been reproduced in ulterior pooled analyses of phase III trials [16] with comparable effect sizes (the EMEA/H/C/572/II/26 procedure reported a 25% decrease in oral clearance in individuals between 29 and 69 years old). These early phase I studies demonstrated increased systemic exposure to duloxetine in the elderly population.

Taken together, the clinical decision of using duloxetine from clinical trials and pharmacokinetic results remains challenging. At present, as the EMA and FDA reports assessing the safety of duloxetine did not find quantitative differences in tolerability between old and non-elderly, no adaptation of dosage is advised in the elderly by the authorities. However, these conclusions rely on post-hoc subgroup analyses with small sample sizes of elderly participants [16], which might reduce the probability of finding a difference in tolerability. However, data is available to assess this tolerability more robustly. As mentioned in the FDA CYMBALTA© Label [17] (revised version of 2017), of the 6781 patients in premarketing clinical studies of duloxetine in all conditions, 15.6% were aged 65 years or over. Although it represents approximately 1058 subjects, no direct comparison of between-age tolerability across conditions has been found in the literature.

Altogether, it seems that a direct evaluation of tolerability of duloxetine in elderly individuals needs clarification to help its prescription. Therefore, we planned to determine whether the use of duloxetine across its approved conditions is associated with a higher risk of both serious adverse events (SAEs) and non-serious adverse events (nsAEs) in elderly adults in comparison to non-elderly, in face of its efficacy and change in quality of life.

### Objectives

Our main objective is to compare the incidence rates of SAEs under duloxetine in comparison to placebo between participants aged 65 or older and non-elderly between 18 and 65 years old, in RCTs for EMA- and FDA-approved conditions. Our primary hypothesis is that the elderly population has higher incidence rates of SAEs caused by duloxetine than non-elderly.

Our secondary objectives are to compare the incidence rates of nsAEs caused by duloxetine between participants at least 65 years of age and non-elderly and to compare both the clinical efficacy of duloxetine on clinical scales and quality of life between elderly individuals and non-elderly across conditions. The outcomes will be evaluated separately for each indication. Furthermore, efficacy and tolerability will be compared between younger elderly (aged between 65 and 75 years) and older elderly (aged 75 years or older) participants.

## Methods and design

This systematic review and meta-analysis of individual participant data (IPD) will be conducted in interventional, prospective, double-blind, randomized, placebo-controlled trials with an independent duloxetine arm. We will conduct a systematic literature review of relevant trials performed on adult participants aged < and ≥ 65 years old in a population suffering from a condition having EMA or FDA approval for duloxetine. The anticipated end date of the study is November 2023.

### Protocol registration and reporting information

The study protocol has been registered within PROSPERO (registration number: CRD42019130488) and is being reported in accordance with the reporting guidance provided in the Preferred Reporting Items for Systematic Reviews and Meta-Analyses Protocols (PRISMA-P) statement [18] (see checklist in Additional file 1). The planned analysis will be reported according to the PRISMA Extension for meta-analysis of individual participant data [19].

### Eligibility criteria

We will use the following eligibility criteria:

- Types of studies: Double-blind RCTs with only adult participants (≥ 18 year old). Each study should include both subjects aged 65 years old or more and participants younger than 65;
- Types of participants: subjects suffering from a disorder with a known approval for duloxetine by the FDA and the EMA, namely, depression, anxiety, diabetic neuropathic pain, fibromyalgia, chronic musculoskeletal pain and stress urinary incontinence;
- Types of interventions: duloxetine regardless of the dosage, administration frequency, and route of administration;
- Type of comparator: placebo;
- Types of outcome measures: report of SAEs for each participant under duloxetine and placebo arms and/or nsAEs and/or efficacy and/or quality of life.

The research will be restricted to trials written in English, regardless of their publication status (published/unpublished).

### Information sources

Searches will be conducted in PubMed (to identify individual studies from published systematic reviews and meta-analyses of duloxetine), ClinicalTrials.gov, Clinicaltrialsregister.eu, data sharing platforms (ClinicalStudyDataRequest.com, YODA and Vivli), FDA drug approval packages and European public assessment reports and withdrawn applications from the EMA website. The review will be performed on studies available in electronic databases from their date of inception to 31 March 2022.

### Search strategy

Trial identification will be systematic with different search strategies depending on the source. First, we will search PubMed for all systematic reviews and meta-analyses involving duloxetine for an approved indication. Then, individual trials will be identified from these systematic reviews and meta-analyses. The search terms will be as follows: “(Duloxetine AND Meta-Analysis[ptyp])”. In ClinicalTrials.gov, the search will be restricted to all interventional studies with adults and older adults, using “duloxetine” as the search term. In Clinicaltrialsregister.eu and in data sharing platforms, the search term will be “duloxetine” with no filter applied. On the FDA website, FDA drug approval packages will be downloaded from the FDA approved drug product, entering “duloxetine” as the search term. On the EMA website, European public assessment reports and withdrawn applications will be selected, which will be limited to reports on humans with no restriction on the authorization status and using “duloxetine” as the search term.

### Study records

#### Data management, selection and collection processes

Selection and coding of the different study characteristics will be performed by two independent reviewers (JCR and AJ) in a blinded manner. A third reviewer (FN) will arbitrate in case of disagreement. Studies appearing to duplicate authors, treatment comparisons, sample sizes and outcomes will be checked one against another to avoid double-counting and integrating data from several reports on the same study and in contact with the study sponsors. A data extraction sheet based on the Cochrane Handbook for Systematic Reviews of Interventions guidelines will be developed. In case of missing data, the sponsor of the study and/or corresponding authors will be contacted.

#### Collecting IPD

A data sharing request will be send to all sponsors for which trials were spontaneously available on data sharing platforms. For the remaining studies, the request will be send to all corresponding authors, and if possible, a research proposal will be address to each pharmaceutical sponsor on data sharing platforms (for Eli Lilly, Shionogi, Pfizer and AbbVie trials) or on the sponsor website (for Lundbeck, Takeda and Merck Sharp & Dohme trials). For willing collaborators, the terms of the collaboration will be specified in a data transfer agreement, signed by representatives of the data provider and of the recipients (Clinical Investigation Center, Department of Clinical Pharmacology, Rennes University Hospital, France).

The requested participant characteristics are baseline age, gender, intervention arm, duloxetine dose, duration of treatment, number of SAEs from baseline to endpoint, number of nsAEs from baseline to endpoint, study primary outcome and its values at baseline and at endpoint, type of quality of life scale used and its values at baseline and at endpoint. Data will be accepted in any suitable electronic format. Checks on the data will be made to ensure data are correctly coded, missing data are correctly identified, extreme values are genuine and data are consistent with published results. Data from all trials will be incorporated into a single database with fields that are consistent across trials.

From a preliminary study selection up to May 2019, a data sharing agreement with Vivli has been contracted the 24 September 2020, but no IPD has been analysed. If new references are identified with the updated search, a new data sharing request will be completed.

### Outcomes

The main outcome is the number (count) of SAEs for each individual patient. In our study, SAE refers exclusively to any undesirable experience associated with the use of a medical product that results in death, is life-threatening, requires hospitalization or prolongation of existing hospitalization, results in persistent or significant disability or is a congenital anomaly or birth defect. This outcome has the advantages of being simple to interpret and clinically relevant for safety estimation, containing a severity criterion from its definition.

Additionally, the following secondary outcomes will be assessed:

- The number of nsAEs: nsAE refers to any untoward medical occurrence in a clinical trial subject administered a medicinal product that does not have severity criteria for a SAE or necessarily has a causal relationship with this treatment. In a randomized placebo-controlled setting, this outcome provides a global estimate of treatment safety, without the severity feature of the SAE.
- The efficacy on the clinical scale for each indication (i.e. depression, anxiety, pain, and urinary incontinence): different scales with the same indication will be standardized by *z*-scores. This outcome will evaluate the clinical benefit in patients.
- The quality of life scores for each indication: the different scales will be standardized by *z*-scores. In studies where multiple quality of life scales were used, a hierarchy will be established by selecting scales that both resume health data in one unique total score and that were the most used among the duloxetine trials. If the only available instrument in a trial did not permit us to resume data into one score (e.g. SF36), its general health subscale was retained as an indicator of quality of life. Quality of life scores yield a broad and ecological estimate of the well-being state of the patient.

### Data synthesis

#### Qualitative synthesis

For each trial, the following will be described:

The characteristics of the study: year, country, number of arms with duloxetine and placebo, funding sources, conflict of interest, and condition;

The characteristics of trial participants: mean age (and its standard deviation), percentage of male and female patients, number of patients included in the analysis, and population of analysis used in the identified report (intention to treat, per protocol, other);

The type of administration, dose and duration;

The outcome measures as stated above (including the exact definition of outcome (e.g. MedDRA or other)).

These data will be summarized in a table.

#### Criteria for IPD synthesis

Every trial with shared individual participant data will be selected for IPD analysis. Missing trials will be explicitly reported with their characteristics and results described.

#### IPD synthesis

We will report the incidence rate ratio separately for elderly and non-elderly and then test the interaction between age and intervention using a random effect meta-analysis. If all data are not directly downloadable together but rather provided remotely on separate interfaces, we will use a two-stage procedure [20] to derive the incidence rate ratio (for counts of binary outcomes) and mean differences (for quantitative outcomes). The first stage consists in comparing the number (count) of SAEs between groups of participants arranged by age using a generalized linear mixed model with a quasi-Poisson link function in each study. The age of each participant (binary variable between “non-elderly” participants between 18 and 65 years old, and “elderly” participants ≥ 65 years old) and his intervention arm (binary variable between participants under duloxetine and participants under placebo) will be considered fixed effects, and the study as a random effect. In the second step, we will pool the extracted incidence rate ratios using a random effect meta-analysis. If all trials IPD are downloadable via the same platform, we will only perform a one-stage meta-analysis.

Similarly, the number of nsAEs (count) will be analysed using a two-step or one-step approach based on a generalized linear mixed model with a quasi-Poisson link function. For both SAEs and nsAEs, we will explore if the interactions between age and intervention are different across the different conditions (major depressive disorder, generalized anxiety disorder, diabetic peripheral neuropathic pain, fibromyalgia, chronic musculoskeletal pain and stress urinary incontinence). For these analyses, a generalized linear model with a quasi-Poisson link function will be used wherein SAE or nSAE will be the dependent variables, the age, intervention and conditions will be considered as fixed effects and the study as random effect. Interactions between those fixed effects will be estimated.

For the analyses of efficacy and quality of life, clinical (i.e. depression, pain, and chronic anxiety scales) and quality of life scales will be analysed separately using a one-stage or two-stage approach relying on a linear mixed model with the same independent variables as for SAE and nSAE. As various scales might be used across studies, the different scales will be first standardized by *z*-scores. The scores of these scales at the last evaluation of the study will be extracted for the analyses. Similar to adverse events, meta-regression by conditions will be performed with age, intervention arm and conditions as fixed effects and the study as random effect.

The proportion of total variability due to between-study heterogeneity and statistical heterogeneity of the effects will be estimated using respectively the *I*^2^ and tau values.

Missing data will be handled by multiple imputation.

#### Sensitivity analyses

A sensitivity analysis will be performed considering the occurrence of SAEs in binary (Yes vs. No) with the one or two-stage approach relying on a generalized linear mixed model with a logistic link function in each individual study.

In another analysis, the effect of age on safety, efficacy and quality of life will also be evaluated using age as a continuous variable.

#### Subgroup analyses

A subgroup analysis will be performed applying the same methodology as for adverse events, efficacy and quality of life, to explore differences between younger elderly (between 65 and 74 years old) and older elderly participants (≥ 75 years old) in terms of adverse events (serious and non-serious), efficacy and quality of life.

#### Risk of bias in individual studies

Two researchers (JCR and AJ) will assess each trial for risk of bias independently, addressing randomization, allocation concealment, blinding of assessors and of study participants, completeness of outcome assessment, selective reporting and other potential sources of bias according to the Cochrane Collaboration tool for assessing risk of bias in its current version, RoB2 [21], at the study level. Discrepancies will be resolved by consensus.

#### Confidence in cumulative evidence

We will use GRADE to rate the overall certainty (quality) of evidence, which includes the evaluation of risk of bias, inconsistency, indirectness, imprecision and publication factors [22].

## Discussion

The scope of this systematic review and individual participant data meta-analysis is intended to inform clinical decision-making for a wide range of conditions for the elderly population. It will provide an overview of the available evidence of the safety of use of duloxetine in the elderly in comparison to non-elderly. We will use a transparent and rigorous procedure to identify and analyse all relevant randomized controlled trials published and unpublished.

There may be some limitations to this systematic review. First, our review might not be totally exhaustive for published articles as we limited this part of the research from published meta-analyses and reviews identified on the PubMed database. In addition, we selected only articles written in English. However, as we will search for all registered trials from regulatory sites, this research strategy should be sufficient to gather all the trials used by the medical regulatory authorities to evaluate the safety of use of duloxetine in adult population. Second, the number of studies which will be included in the analysis depends on the authorization of their sponsors. While we are using a large set of studies across many conditions, the incidence of serious adverse events may be rare, and we might lack of power to find a higher risk of SAE in elderly population compared to non-elderly adults.

The dissemination plan is to publish results in a peer-reviewed academic journal.

Any amendments made to this protocol when conducting the study will be outlined and reported in the final manuscript.

## Supplementary Information

**Additional file 1.**

## Data Availability

We will publish the codes in the supplementary material of the systematic review. No individual participant data will be published as contracted with the data sharing platform.

## Abbreviations

CI: Confidence interval
EMA: European Medical Agency
FDA: Food and Drug Administration
IPD: Individual participant data
nsAE: Non-serious adverse event
OR: Odds ratio
RCT: Randomized controlled trial
SAE: Serious adverse event
SF36: 36-Item Short Form Survey
